# Indications for late preterm birth, and factors associated with short term maternal and neonatal outcomes at a tertiary care institution

**DOI:** 10.4314/ahs.v22i4.75

**Published:** 2022-12

**Authors:** Deeshah M Deelchand, Thinagrin D Naidoo

**Affiliations:** Department of Obstetrics and Gynaecology, Grey's Hospital, Pietermaritzburg and University of Kwazulu-Natal, South Africa

**Keywords:** Late preterm birth, indications, hypertensive disorder of pregnancy, short term maternal outcomes, short term neonatal outcomes, factors

## Abstract

**Background:**

The preterm birth rate is rising mainly because of the marked increase in late preterm deliveries.

**Objectives:**

To evaluate the indications for LPTB and the factors associated with the short term maternal and neonatal outcomes.

**Methods:**

This retrospective study was conducted at a tertiary health care institution. The study sample included 191 women who delivered between October 2019 to November 2020.

**Results:**

The majority (81%) were medically indicated LPTB, and mainly for maternal indications (77%). The most common maternal indication for LPTB was for hypertensive disease of pregnancy (HDP) (82.5%). There was a significant increase in the high care/ ICU admission for maternal indication of LPTB, maternal age < 20 years, and patients with HDP. There was 1 maternal death and 1 neonatal death. 48% of the neonates were admitted to NICU and 53% had neonatal complications. Neonates born by caesarean delivery were more likely to have respiratory complications and be admitted to NICU.

**Conclusion:**

These maternal/ neonatal factors should be used to identify patients at risk of adverse maternal and neonatal outcomes.

## Introduction

'According to WHO, an estimated 15 million babies are born preterm (before 37 weeks of pregnancy) every year’. In resource constrained countries, on average, 12% of babies are born preterm compared with 9% in well-resourced countries. The rate of preterm birth in South Africa increased from 10% in 2010 to 12.4% in 2014.^1^ Late preterm births (LPTB) accounted for approximately 73% of all preterm births and about 7.4% of total births in the United States in 2020.^2^ The preterm birth rate is rising mainly because of the marked increase in late preterm deliveries (between 34 °/7 and 36 6/7 weeks).^3^

Studies have highlighted increased morbidity and mortality associated with LPTB.^4^, ^5^ McIntire et al.^4^ concluded that ‘neonatal mortality rate was higher in the LPTB compared with term deliveries and neonatal morbidity was significantly increased at 34–36 weeks. Shapiro-Mendoza et al. also found that ‘late preterm infants were seven times more likely to have newborn morbidity than term infants (22% vs 3%)’. 5

In South Africa more than 8 out of every 100 babies are born preterm and the country has been ranked 24 out of 184 countries for the number of newborn deaths due to complications of prematurity. 6 However, there are no known local studies or studies done in other resource constrained settings that look specifically at the indications of LPTB and factors associated with their outcomes. Research into the causes of late preterm birth and the factors associated with the outcomes with regard to the mother and the neonate will help in developing interventions to predict and decrease the rate of late preterm births and mitigate the impact of adverse maternal and neonatal outcomes.

The aim of this study was to evaluate the indications for late preterm birth and the factors associated with the short-term maternal and neonatal outcomes.

## Methods

This retrospective study was conducted at a tertiary health care institution (Grey's Hospital) in Pietermaritzburg in the province of Kwazulu-Natal, South Africa from October 2019 to November 2020. Data was collected by means of a data collection tool which included maternal demographics, clinical characteristics, index pregnancy, medical comorbidities, intrapartum details and maternal outcomes postpartum. It also included the neonatal characteristics and outcomes. All live singleton late preterm births were included using the labour ward registry which contains the records of the patients who delivered. The maternity and neonatal files were retrieved for data collection. The indication for delivery included spontaneous or indicated delivery. Spontaneous delivery is defined as any birth after preterm labour or preterm premature rupture of membranes (PPROM) in the late preterm period. Indicated delivery is defined as induction of labour or caesarean section for maternal or foetal reasons. Gestational age was determined according to the best obstetric estimate of the attending obstetrician based on the last menstrual period and the early ultrasound. Institutional ethical and regulatory permission was obtained for the study (UKZN; BREC/00001112/2020).

### Statistical analysis

The data was analysed using IBM SPSS Statistics version 22 (IBM, Armonk, NY, USA). The Pearson chi-square test determined if there were statistically significant associations between the independent variables in the study and the short term maternal and neonatal outcomes. Independent variables that were found to be significantly associated with the maternal and neonatal outcomes were compared using binary logistic regression analysis to determine if they were significant predictors of the outcomes. The logistic regression results are reported as odd ratios for the respective maternal and neonatal outcomes. Significance testing was set at the 95% confidence. P value <0.05 was taken as statistically significant.

## Results

A total of 1561 women delivered from October 2019 to November 2020, of which, 291 women had late preterm deliveries. One hundred patients were excluded. The final study sample included a total of 191 women who had late preterm births, and their neonates who were delivered by vaginal delivery or caesarean delivery.

**Figure:**
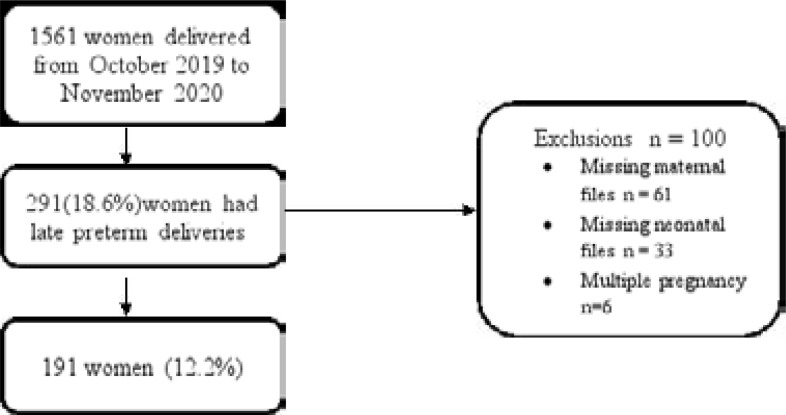


### Study sample

The majority of the women who delivered were primigravida's (35%), and 26% had a previous caesarean delivery. Seventy two percent of the late preterm births were complicated by hypertensive disorder of pregnancy of whom 72% had preeclampsia with severe features, 7% had chronic hypertension with superimposed preeclampsia with severe features, 9% were eclamptic, 4% had preeclampsia, 4% had gestational hypertension, 5 % had chronic hypertension. Of the 5% who had renal disease, 56% had acute kidney injury. Of the 5% diagnosed with cardiovascular disease, 22% had mitral valve disease while 11% had prosthetic valve replacements and 33% had heart failure. (Table 1)

**Table 1:** Maternal comorbidities

Comorbidities	Subcategory	n (%)
Anaemia		36 (19)
Diabetes	Gestational diabetes Overt diabetes	6 (3) 3 (1.6) 3(1.6)
Hypertension	Chronic hypertension Gestational hypertension Pre-eclampsia Chronic hypertension with superimposed preeclampsia with severe features Preeclampsia with severe features Eclampsia	138 (72) 7 (5) 6 (4) 5 (4) 9 (7) 99 (72) 12 (9)
CNS disease	Epilepsy Other	4 (2) 3 (1.6) 1 (0.5)
Renal disease	Nephrotic syndrome AKI Hydronephrosis/ recurrent pyelonephritis	9 (5) 2 (1) 5 (2.6) 2 (1)
Pulmonary disease	Pulmonary Tuberculosis Lower respiratory tract infection/ pneumonia Atelectasis	4 (2) 1 (33) 1 (33) 1 (33)
Auto-immune disease	SLE RA Other	5 (3) 1 (0.5) 1 (0.5) 3 (1.6)
Cardiovascular disease	Mitral valve disease Prosthetic valve replacement Heart failure Other	9 (5) 2 (22) 1 (11) 3 (33) 3 (33)

The LPTB rate was 18.6%, and the majority (81%) were medically indicated, mainly for maternal indications (77%), 19% had spontaneous LPTB. The most common maternal indication for late preterm deliveries was HDP (82.5%). Regarding deliveries for foetal indications, 54.2% were delivered for a non-reassuring foetal heart rate, 28.5% for foetal growth restriction, 11.4% for abruptio placenta grade 2, while 5.7% of deliveries were for congenital anomalies. 85% of the patients underwent caesarean deliveries, while 1% underwent exploratory laparotomies for advanced abdominal pregnancies. The maternal and foetal indications for LPTB are depicted in the table below.

Postpartum complications occurred in 10% of mothers who delivered, with 22% having postpartum haemorrhage (PPH), and 22% requiring mechanical ventilation. 81% of the entire cohort were admitted for < 5 days. Half of the admissions went to the High Care Unit (HCU) (51%) and 4% of the patients went to the Intensive Care Unit (ICU). Empirical use of antibiotic was prescribed for 10% of the women postpartum. There was 1 maternal death during the study period secondary to non-pregnancy related infection as depicted in Table 3.

**Table 3:** Short term maternal outcomes

Maternal outcomes	Sub- category	n (%)
**Postpartum complications**	PPH Relaparotomy Heart failure Puerperal sepsis Mechanical ventilation Respiratory distress Wound hematoma Postpartum psychosis	20 (10) 4 (22) 1 (6) 1 (6) 2 (11) 4 (22) 3 (17) 1 (6) 2 (11)
**Admission postpartum**	Postnatal ward HCU ICU	86 (45) 98 (51) 7 (4)
**Number of days admitted postpartum**	<5 days 5–10 days >10 days	154 (81) 28 (15) 9 (5)
**Maternal transfusion postpartum**		12 (6)
**Postpartum use of antibiotic**		20 (10)
**Maternal death**		1 (0.5)

Of the 191 neonates, 6% had an Apgar score of < 7 at 5 minutes. 35% of the neonates required resuscitation at birth and 48% were admitted to NICU. Complications occurred in 53% of neonates of which 39% had respiratory complications, 33% had metabolic complications; including hyperbilirubinemia (74%), hypoglycaemia (21%), and neurological (5%)- the most common being secondary to neonatal encephalopathy (70%). 37% of the neonates were admitted for < 5 days compared to 36% who were admitted for between 5–10 days. There was 1 neonatal death secondary to non-viable congenital anomaly as shown in Table 4.

**Table 4:** Short term neonatal outcomes

Neonatal Outcomes	Subcategory	n (%)
Apgar at 5 min	>=7 <=6	180 (94) 11 (6)
Resuscitation at birth		67 (35)
Admission to NICU		92 (48)
Number of days in NICU	< 5 days 5–10 days >10 days	34 (37) 33 (36) 25 (27)
Neonatal complications		102 (53)
Respiratory complications		75 (39)
Metabolic complications		63 (33)
Neurological complications		10 (5)
Gastrointestinal complication		20 (10)
Infection		22 (12)
Neonatal death		1 (0.5)

A maternal age 30–34 years was found to be 7 times more likely to be associated with late preterm delivery (p = 0.03, OR 6.9 (1.3–37.4)). Hypertensive patients in this study were more likely to be delivered for a maternal indication. (p < 0.001, OR 6.4 (2.4–17.0)). There was a statistically significant increase in the HCU / ICU admission for maternal age <20 years, and hypertensive disorder of pregnancy as shown in Table 5.

**Table 5:** Variables associated with indications of late preterm births and short term maternal and neonatal outcomes.

**Variables associated with** **maternal indication for late** **preterm births**	**n (%)**	**OR (95% CI)**	**p value**
Maternal age 30–34 years Hypertension	26 (13.6) 120 (62.8)	6.9 (1.3 – 37.4) 6.4 (2.4 – 17.0)	0.03 < 0.001
**Variables associated** **with postpartum admission to** **HCU/ ICU**	**n (%)**	**OR (95% CI)**	**p value**
Maternal indication for late preterm births Maternal age <20 years Hypertension	120 (62.8) 31 (16.2) 138 (72.2)	5.3 (2.0 – 14.0) 2.6 (1.2–5.8) 2.9 (1.2–7.1)	<0.001 0.01 <0.001
**Variables associated with** **NICU admission**	**n (%)**	**OR (95% CI)**	**p value**
Caesarean Delivery	163 (85.3)	2.8 (1.1–7.1)	0.02
**Variables associated** **with neonatal respiratory** **complications**	**n (%)**	**OR (95% CI)**	**p value**
Caesarean Delivery	163 (85.3)	3.0 (1.1–8.4)	0.03

Rate of NICU admission was found to be significant when compared with mode of delivery by caesarean section for late preterm births. (p=0.02, OR=2.8, 95% CI=1.1–7.1). Neonates who were delivered by caesarean section were more likely to have respiratory complications. (p=0.03, OR=3.0, 95% CI=1.1–8.4) as depicted in Table 5. Variables that showed a significant association with late preterm birth, short term maternal and neonatal outcomes have been included in Table 5.

## Discussion

In this study, we sought to evaluate the indications for late preterm births and the factors associated with the short term maternal and neonatal outcomes in our institution. We found that the rate of LPTB in our setting was higher when compared to a well-resourced country like the United States where it was 7.4% in 2020.^2^ This could be explained by the fact that our institution is a tertiary referral centre for high-risk obstetrics patients. Our findings however are similar to that of Blencowe et al. who showed that ‘the rate of preterm birth was higher in developing countries compared to developed countries ranging from about 5% in several European countries to 18% in some African countries in 2010.^7^

In our study the majority of the LPTBs were due to medical indications as compared to spontaneous delivery. From the iatrogenic deliveries, maternal indications vastly outweighed fetal indications for delivery. This is in contrast to the study by Laughon et al. that reported that only 35% of the cohort for late preterm birth was medically induced while 65% had spontaneous onset of late preterm delivery 8.

Singh et al showed in his study in India that ‘HDP (21.1%) was significantly associated with increased incidence of LPTB as compared to early term births’.^9^ We have demonstrated similar findings whereby HDP was the most common maternal indication for LPTB. Non-reassuring FHR followed by FGR were found to be the most common foetal indications for LPTB in our study. Similarly in the study by Carreno et al, he found that ‘FGR complicated approximately one third of all cases of medically indicated LPTB’.^10^ We also found in our study that women aged 30–34 years were more likely to be delivered for maternal indication for LPTB. This is in contrast to the study by Lu et al. who found that mothers younger than 20 years or older than 35 years of age were one of the most important factors associated with the delivery of late preterm infants. 11 Younger mothers and those with HDP were also found to be more at risk for admission to the high-risk obstetric unit. There are no known local studies of the factors associated with the maternal outcomes of LPTB. The high rate of NICU admission in this study can be compared to the study by Lubow et al. who showed that ‘late preterm infants had higher rates of neonatal intensive care unit (NICU) admissions (56% vs 4%) when compared to term infants’.^12^ Neonates who were delivered by caesarean section were found to more likely to have respiratory complications. Similarly in the study by Jain et al, ‘the risk of respiratory distress secondary to transient tachypnea of the newborn, surfactant deficiency, and pulmonary hypertension is increased’.^13^ The rate of NICU admission was also found to be significant when compared with mode of delivery by caesarean section for late preterm births. Villar et al also showed that ‘elective caesarean delivery without labour was associated with an increased risk for admission to NICU for seven or more days. 14 Late preterm delivery was not the attributable cause of maternal and neonatal death in this study. The limitations of our study include the fact that it was a retrospective study. Maternal and neonatal outcomes were only assessed until discharge. It was a single centre study. Neonatal morbidity in the late preterm period will also be affected by the maternal medical conditions. One of the strengths of the study was that it was conducted at a single institution whereby a standardised management of care is practiced for all patients. It was also the first known local study on the indications of LPTB and the factors associated with the outcomes.

## Conclusion

The findings of this study demonstrate that the rate of late preterm birth in our setting is higher when compared to well resource countries. The main maternal indication for late preterm birth was HDP while a non-reassuring foetal heart rate was the most common fetal indication for delivery. We also found that older mothers and HDP were risk factors for medically indicated LPTB. Younger mothers, patients with HDP and those delivered for a maternal indication were more at risk for admission postpartum to the high-risk obstetric unit. Also, neonates born by caesarean delivery were more likely to be admitted to NICU and to have respiratory complications. In conclusion, these maternal/ neonatal factors should be used to identify patients at risk of adverse maternal and neonatal outcomes. Targeting the risk factors for LPTB could over the long run decrease the rate of LPTB and the maternal and neonatal morbidity. Long term follow up of the mothers and neonates is required to further understand the burden of the risks on them in terms of morbidity and mortality. Well-designed population representative cohort studies from multiple centres in South Africa that will evaluate the indications of LPTB and the factors associated with the maternal and neonatal outcomes are thus needed to address PTB data gaps in these settings as LPTB forms a major part of the PTB.

## Figures and Tables

**Table 2:** Indications for indicated late preterm births

Maternal indications for late preterm births	n (%) 120 (77%)	Fetal indications for late preterm births	n (%) 35 (23%)
HDP	99 (82.5)	Non reassuring fetal heart rate	19 (54.2)
Placenta previa	9 (7.5)	FGR	10 (28.5)
Renal	2 (1.6)	Abruptio placenta	4 (11.4)
Cardiac	1 (0.8)	Congenital anomalies	2 (5.7)
CNS	1 (0.8)		
Previous CD in labour	1 (0.8)		
Others	7 (5.8)		
